# Smartphone Delivery of Mobile HIV Risk Reduction Education

**DOI:** 10.1155/2013/231956

**Published:** 2013-09-17

**Authors:** Karran A. Phillips, David H. Epstein, Mustapha Mezghanni, Massoud Vahabzadeh, David Reamer, Daniel Agage, Kenzie L. Preston

**Affiliations:** National Institute on Drug Abuse, Intramural Research Program, National Institutes of Health, 251 Bayview Boulevard, BRC Building, Suite 200, Baltimore, MD 21224, USA

## Abstract

We sought to develop and deploy a video-based smartphone-delivered mobile HIV Risk Reduction (mHIVRR) intervention to individuals in an addiction treatment clinic. We developed 3 video modules that consisted of a 10-minute HIVRR video, 11 acceptability questions, and 3 knowledge questions and deployed them as a secondary study within a larger study of ecological momentary and geographical momentary assessments. All 24 individuals who remained in the main study long enough completed the mHIVRR secondary study. All 3 videos met our a priori criteria for acceptability “as is” in the population: they achieved median scores of ≤2.5 on a 5-point Likert scale; ≤20% of the individuals gave them the most negative rating on the scale; a majority of the individuals stated that they would not prefer other formats over video-based smartphone-delivered one (all *P* < 0.05). Additionally, all of our video modules met our a priori criteria for feasibility: ≤20% of data were missing due to participant noncompliance and ≤20% were missing due to technical failure. We concluded that video-based mHIVRR education delivered via smartphone is acceptable, feasible and may increase HIV/STD risk reduction knowledge. Future studies, with pre-intervention assessments of knowledge and random assignment, are needed to confirm these findings.

## 1. Introduction

The use of mobile and desktop computer technologies in HIV healthcare and prevention delivery has been on the rise using a variety of technology platforms, including desktop computers [1–4], web-based systems [5–8], social networking sites, interactive voice response [9], personal digital assistants (PDAs)/smartphones, and short message service (SMS)/text messaging [10–13]. The range of indications for these electronic interventions is even broader than the range of technologies used; electronic interventions have been explored for HIV prevention [2], self-efficacy enhancement [14], antiretroviral therapy adherence [7], social support, appropriate care referrals [1], and internet health literacy [15].

The interest in electronic technology in healthcare delivery derives in large part from its potential to increase access to care in a cost-effective manner, especially for people who are underserved due to poverty, rural residence, unforgiving schedules, or other barriers to regular office visits. There is growing evidence that mobile health technologies can be effectively utilized in resource-limited settings in both the developed and developing world. For example, Muessig et al. [16] found that for young black men who have sex with men (MSM) in North Carolina, mobile technologies were widely used as an acceptable means of HIV intervention. Winstead-Derlega et al. found that it was feasible and acceptable to use mobile media to deliver peer health messages to HIV-positive adults in rural Virginia [17]. In a randomized study in Kenya, Lester et al. found that patients who received a mobile phone SMS intervention had significantly improved adherence to antiretroviral therapy (ART) compared with those who received standard of care [18]. Additional benefits of electronic intervention include the potential for the immediate delivery of care in the participant's natural environment with reduced needs for space, staff, and training. 

Evidence is mounting for the effectiveness of electronic delivery of health information relevant to HIV risk reduction. A 2012 meta-analysis of 15 randomized controlled trials showed that compared to minimal intervention, interactive computer-based interventions had significant effects on sexual health knowledge, safer sex self-efficacy, safer sex intentions, and sexual behavior [14]. Improved dissemination of HIV prevention education related to intravenous drug use and sexually transmitted disease has been demonstrated for computer-based [16] and video-based [17] programs. Some studies even suggest that privacy during the electronic delivery of the HIV education is preferred [16], though the addition of an interactive group session can enhance the benefits of the videos [19]. Person et al. surveyed over 300 individuals with HIV, latent TB, or who were being screened for HIV, TB, or syphilis and found that cell phones and text messaging were prevalent and receptiveness to text messaging for healthcare-related communication was high [20].

The widening use of smartphones promises to enhance the disseminability of mobile health education videos. Jones et al. [21–23] tested the use of smartphones for the delivery of HIV prevention messages to women in a randomized clinical trial, comparing 12 weekly videos of the educational soap opera *Love, Sex, and Choices* to 12 weekly HIV prevention messages. Baseline and post-intervention interviews at 3 and 6 months were completed by an audio computer-assisted self-interview (ACASI). At baseline, 99% of the participants reported having unprotected vaginal sex and 44% reported having unprotected anal sex with high-risk partners. Both intervention groups reported a significantly reduced risk post-intervention (*P* < 0.001); the magnitude of reduction did not statistically significantly differ by group; *P* = 0.23. However, after adjusting for baseline sexual activity, women receiving the video intervention had roughly a 20% greater reduction in risk behavior. The authors concluded that both smartphone interventions were viable for HIV prevention [21–23].

Our goal was to develop and deploy via smartphone an interactive mobile HIV Risk Reduction (mHIVRR) education intervention and determine via smartphone-delivered assessments whether it reduces HIV/STD-related risk via increased HIV/STD knowledge. For our intervention, we selected *Safe in the City, *an STD/HIV prevention video geared for patients in an STD clinic waiting room that was shown to decrease STDs in a controlled clinical trial [24]. *Safe in the City* is distributed in user-friendly kits as a part of the Diffusion of Effective Behavioral Interventions (DEBI) project of the Centers for Disease Control and Prevention (CDC). We evaluated the feasibility and acceptability of *Safe in the City* modified for “on demand” delivery on a handheld device in opioid-dependent patients in methadone maintenance therapy. Our mHIVRR modules consisted of 3 components: (1) the videos themselves, (2) questions about their acceptability to the user, and (3) questions about their perceived effectiveness for the user.

## 2. Materials and Methods

### 2.1. Study Participants

 This study was conducted as part of a larger 46-week natural history study of personal and environmental stress and drug use, the goal of which is to develop field-deployable measures of environmental influences (stressors, drug exposure, etc.) that could ultimately be used in studies of gene-environment interactions. Participants in the main study were opioid-dependent polydrug users recruited by newspaper and word of mouth. They received opioid agonist treatment (OAT) with either methadone or buprenorphine, based on their preference, and weekly individual counseling. Inclusion criteria included age between 18 and 75, physical dependence on opioids, and residence in or near Baltimore. Study exclusion included history of any DSM-IV psychotic disorder, bipolar disorder, or current Major Depressive Disorder; current dependence on alcohol or sedative hypnotics; cognitive impairment severe enough to preclude informed consent or valid self-report; and medical illness or medications that affect the hypothalamic-pituitary-adrenal (HPA) axis. As part of the main study, participants provided thrice weekly urine for toxicology and were issued a smartphone and GPS to track drug use, stress, and geographical location (a measure of environmental risk) for 16 of the first 18 weeks of the main study. They were allowed one smartphone replacement for a lost, stolen, or damaged unit. If a second unit was lost, damaged, or stolen, the participant was withdrawn from the main study and transferred to OAT in the community. At the end of the main study, participants were given the option to either keep the smartphone or return it and receive $100. 

For recruitment into the present mHIVRR secondary study, participants in the main study were asked in person by investigators to participate once they reached week 7 of the main study. Participation in the main study was not affected by their decision to participate or not in the mHIVRR secondary study. This study was approved by the Institutional Review Board of the NIDA Intramural Research Program, included a Certificate of Confidentiality, and each participant gave written informed consent. 

After giving written informed consent for the mHIVRR study, participants were given a 5-minute demonstration on how to initiate a module and answer questions. Participants were asked to view a video and answer the subsequent questions at least once during the week but could watch it as many times as they chose. Participants met weekly with investigators, at which time new modules were downloaded onto the smartphone and the data uploaded from the smartphone. Uploaded data included logs of the number of times video viewings were attempted and completed, the percentage of the video viewed each time, and the responses to the questionnaires. Each time a new module was downloaded onto the phone, the prior week's module remained available for repeated viewing. Participants completed questionnaires on the smartphone after each viewing of ≥75% of an HIVRR video component. If less than 75% of the video was viewed, no questions appeared. Participants were compensated $20 if they watched a module and answered all the questions at least once. Total compensation for completing all 3 video modules at least once was $60.

### 2.2. Video Component

*Safe in the City* is a 23-minute STD/HIV prevention video geared for patients in an STD clinic waiting room. The video contains key prevention messages aimed at increasing knowledge and perception of STD/HIV risk, promoting positive attitudes towards condom use, building self-efficacy and skills to facilitate safer sex, and the acquisition, negotiation, and use of condoms. There are three interwoven vignettes that model negotiating safer sexual behaviors among young couples of diverse racial/ethnic backgrounds and sexual orientations. Animated segments demonstrate proper condom use and the variety of condoms available. We divided the 3 vignettes into 3 separate videos, each about 8–10 minutes including credits and formatted them for use on a smartphone running the Windows Mobile 6 operating system.

### 2.3. Acceptability Questions and Feasibility Assessment

The same 11 acceptability questions appeared on the smartphone after each viewing of ≥75% of the video component in all 3 modules. The acceptability questions were divided into 3 main categories: functional acceptability, educational acceptability, and comparative acceptability (Table 1). The functional acceptability questions assessed the ease of loading and playing the mHIVRR videos on the smartphone. The educational acceptability questions assessed the perceived educational value of the videos. The comparative acceptability questions assessed whether participants would have preferred to have the intervention delivered through a different medium.

To assess the acceptability of the mHIVRR modules, we collected data on four main elements. For questions 1 through 7, element 1: the the median rating on a 5-point Likert scale, with acceptability defined as ≤2.5, and element 2: the percentage of participants rating anything as 5 (“very hard,” “not at all effective,” “not at all applicable,” and “very boring”) with acceptability defined as ≤20%. For question 8, element 3: the percentage of participants giving a rating of 5 (“much too long”) and/or 1 (“nowhere near long enough”), with acceptability defined as ≤20%. For questions 9–11, element 4: the percentage responding “no,” with acceptability being a majority. To assess feasibility, we collected data on two additional elements: (1) data missing due to noncompliance, with feasibility being defined as ≤20%; and (2) data missing due to technical failure, with feasibility being defined as ≤20%. 

### 2.4. Knowledge/Effectiveness Questions

While effectiveness assessment was not the main focus of this nonrandomized study, we did want to get a sense of whether participants were actually learning or reinforcing HIVRR knowledge. Three unique knowledge questions directly related to the video component followed each appearance of the acceptability questions (Table 2). We considered the mHIVRR intervention effective if at least 80% of the participants score >65% “correct” on most modules. 

### 2.5. Other Study Activities

#### 2.5.1. Ecological Momentary Assessment (EMA)

Using the same smartphones utilized in the mHIVRR project, participants initiated entries (1) each time that they used a drug and (2) each time that they felt overwhelmed, anxious, or stressed more than usual. Participants also made 3 random signal-triggered recordings per day and one brief “end of day” recording. 

#### 2.5.2. Urine Toxicology

Participants provided urine samples three times per week. Samples were tested for amphetamines, barbiturates, benzodiazepines, THC, cocaine, methadone, codeine/morphine, and PCP.

#### 2.5.3. Questionnaires and Interviews

The questionnaires and interviews from the main study that were included in this secondary study analysis were the Addiction Severity Index (ASI) [25] and the HIV Risk-Taking Behavior Scale (HRBS) [26]. The ASI was interviewer-administered and completed at baseline prior to entry into the main study. The HRBS, which assesses behaviors associated with an increased risk of HIV infection and includes subscales for drug-related risk (6 questions) and sex-related risk (5 questions), was completed via computer prior to participation in the mHIVRR secondary study. We made two modifications to the HRBS: we added a sixth question to the sex-related risk section inquiring about condom use during anal sex, resulting in two subscales, each with 6 questions, and we changed the timeframe from the last month to the last 2 weeks to match the frequency of questionnaire administration.

### 2.6. Data Analysis

We compared median scores for all functional acceptability and educational acceptability questions using the Wilcoxon rank sum test, a nonparametric alternative to the paired *t*-test, appropriate for ordinal variables. We utilized Fisher's exact test to analyze the comparative acceptability and knowledge/effectiveness questions. We also undertook exploratory analyses for indications that acceptability varied by OAT (methadone versus buprenorphine), sex, or other demographic and drug use variables utilizing the Fisher's exact test and *t*-tests.

Although the study was designed to assess feasibility and acceptability, we also explored the intervention's possible role in behavior change by comparing the percentage of urines positive for heroin and/or cocaine in the 12 urine time points (approximately 1 month) pre-mHIVRR intervention to the 12 urine time points (approximately 1 month) post-mHIVRR intervention utilizing paired *t*-tests and Wilcoxon signed rank tests. We assessed EMA reporting of route of administration of heroin and/or cocaine during these same time frames utilizing the same tests. Analyses were done with Stata 10 (StataCorp LP, 1996–2013).

## 3. Results 

 The demographic and drug use characteristics of our sample are shown in Table 3. All participants approached to participate in this secondary study agreed to participate. Our sample included 26 participants, 24 (92%) of whom completed all 3 mHIVRR modules. Two participants did not complete the mHIVRR study because, while participating in it, they were discharged from the main study as per protocol for missing more than 3 consecutive clinic days without contacting the clinic. At entry into the mHIVRR secondary study, among participants receiving methadone the average dose was 88 ± 23 mg (mean ± SD) and among participants receiving buprenorphine the average dose was 17 ± 4 mg (mean ± SD).

 We assessed HIV risk with the HRBS prior to entrance into the main study. The total risk score was 4.9 ± 4.6 (mean ± SD); there was no difference based on OAT type (methadone versus buprenorphine) (*t* = 0.100, *df* = 16, and *P* = 0.922). The drug-related risk score was 0 (0,1) (median (IQR)) and the sex-related risk subscale score was 2.5 (0,6) (median (IQR)); neither differed by OAT type (*z* = 1.679, *P* = 0.093 and *z* = −1.069, *P* = 0.285, resp.). 

### 3.1. Functional and Educational Acceptability

Acceptability questions 1–3 addressed functionality in regards to being able to play, see/hear, and understand the video modules. Questions 4–7 assessed the perceived educational value of the video modules. Median ratings for questions 1–7 across all videos for all participants were less than or equal to 2.5. There were differences by OAT with the methadone group providing significantly higher (but still less than or equal to a median score of 2.5) ratings on questions 1 (play), 3 (understand), 4 (learn something new), 5 (remind me of something I knew), and 7 (entertaining) (Table 1). 

 In addition to exploring median ratings of functional and educational acceptability, we looked at the percent of participants rating each question a 5 which was the most negative response category on the 5-point Likert scale. A percent rating of 5 for questions 1–7 was ≤20% and consistent with acceptability as we defined  a priori  for all of the questions across all 3 videos. When broken down by OAT, only question 6 (applicability) in the buprenorphine group received a percent rating of >20% (31%) (Table 1). 

 Question 8 addressed the perceived appropriateness of the length of the video modules. All percent ratings for extreme responses (1 or 5 on the Likert scale) for question 8 were ≤20% and in keeping with our definition of acceptability. A percent rating of 1 (“nowhere near long enough”) for question 8 occurred in 4.5% of the responses and 5 (“much too long”) occurred at a rate of 1.3% (Table 1).

### 3.2. Comparative Acceptability

Responses to questions showed that no other medium of delivery was hypothetically preferred over the smartphones. The booklet-based format was not preferred in 87.3% to 91.8% of participant viewings across all 3 videos. The computer-based format was not preferred in 63.3% to 76.4% of participant viewings across all 3 videos. The text-based format was not preferred in 89.1% to 93.9% of participant viewings across all 3 videos. The only difference by OAT was more methadone compared to buprenorphine participants preferred a text-based format (*P* = 0.012) but none of these alternate format preferences achieved a majority which is consistent with our a priori definition of acceptability (Table 1). 

### 3.3. Feasibility

There were no data missing due to participant noncompliance; all 24 participants viewed ≥75% and completed all acceptability and knowledge questions for all 3 videos. There were also no data missing due to technical failure. 

### 3.4. Knowledge/Effectiveness

We met our a priori criterion for module effectiveness (at least 80% of participants scoring >65% “correct” on most modules). Across all 3 videos, all 3 knowledge questions, and all participants, 92% of responses were correct (Table 2).

### 3.5. Urine Toxicology and EMA

 We compared urine results in the 1 month (12 urines) prior to the 4-week mHIVRR intervention to urine results in the 1 month (12 urines) after the intervention period. We found no change in the percent of heroin-positive or the percent of cocaine-positive urines.

 For the same time periods, we compared EMA reports of drug use to determine whether routes of administration of heroin and/or cocaine changed pre- and post-intervention. We found no change in the real-time self-reported route of administration.

## 4. Discussion

This project was in keeping with the suggestion, in a 2012 Cochrane Review, that investigators conduct further research into mobile phone messaging interventions for self-management of long-term illnesses [27]. We noted a high completion rate of 92% for all 3 mHIVRR modules, we met all our a priori criteria for acceptability, and we encountered no technical issues, showing that the use of smartphones to deliver HIV-risk education is both feasible and acceptable in our polydrug-using population. 

Overall, our sample was representative of treatment seekers in Baltimore [28, 29]. Their drug use histories were also similar to those seen in previously published studies and did not differ by OAT medication (with the exception of “other opiate” use in last 30 days, which was higher in the buprenorphine-treated group than in the methadone-treated group).

At baseline, our sample had low overall levels of drug-related risk, sex-related risk, and total risk (previous studies have demonstrated that IDUs' reports of both demographic and HIV risk behavior can be reliable) [30]. That finding is consistent with those of the national Drug Abuse Treatment Outcome Studies (DATOS), which found that treatment programs in cities with higher prevalence rates of HIV/AIDS such as Baltimore admitted clients with lower baseline levels of risk behavior than other cities [31]. In 2011, Chaudhry et al. [32] reported that among 303 buprenorphine-maintained individuals across nine US sites, 24% had had sex without a condom and almost 9% had shared needles in the previous 90 days. In their sample, as in our sample, risk factors for unprotected sex included having a regular partner. Addressing transmission risk behaviors is an important secondary HIV prevention strategy, and, based on our data and those of others, there is still a need for education surrounding needle cleaning and condom use. 

 Our participants' responses to questions on functional and educational acceptability met our a priori criteria for “acceptable as is.” For all 3 videos, there were differences by OAT, with the methadone group having higher (but still ≤2.5) median ratings than the buprenorphine group. Given that demographic and drug use characteristics were similar across the two groups, it is unclear why the methadone group rated the videos more negatively. 

Across all 3 videos, the percentage of participants rating a 5 (most negative response) was ≤20% and consistent with acceptability as we defined it a priori. However, when we broke down the results by OAT and by question, we found that for question 6 (how much did this module apply to situations in your life?), 31% of buprenorphine-maintained participants stated it was “not at all applicable to my life.” The higher negative rating for this question may have been a result of participants' taking the question to refer to whether they had same-sex partners, rather than thinking more broadly about the video's messages regarding condom use and STD testing. Additionally, while participants were assured that the information we gathered was for research purposes only, there may have been a concern regarding the perceived stigma related to same-sex partners or adultery, causing participants to falsify their response to this question. Negative ratings were more frequent in the buprenorphine group in all 3 videos, but there were no significant differences between groups in median ratings or percentages.

 Video module length was rated “just right” by the majority of participants, both for each individual video and for all three videos combined. Several of the videos were watched more than once, further supporting the questionnaire responses on the videos' appropriate length and high functional and educational acceptability.

None of the alternative formats presented in the comparative acceptability questions were preferred over the smartphone-delivered video-based format. These comparisons were hypothetical, however, since the participants were not exposed to the booklet or computer-delivered format during the study. Again, the fact that multiple participants viewed the videos more than the required one time also supports the comparative acceptability of the modules.

Based on the low rates of data missing due to noncompliance and technical failure, we determined that the mHIVRR intervention was feasible. The high compliance rate we achieved in this mHIVRR study may be partly attributed to the fact that participants were participating in a larger EMA study and had already been using the smartphones for at least 7 weeks. We assessed the technical issues by participant interviews at the weekly meetings for data upload and video download. There were viewing initiations that did not reach the 75% completion mark, and by design no acceptability and knowledge questions appeared. These incomplete initiations were expected, as we had provided no way to pause and restart the video, given the sensitive nature of the video topics, in case the smartphone was left unattended or picked up by another individual. According to participant report, the incomplete initiations resulted from social interruptions, not technical difficulties. All smartphones were password-protected to reduce the risk of confidentiality breach; no individuals misplaced their log-in or password information. There were also no issues with recharging the devices, and no smartphones were lost or damaged.

 While not a main focus of the study, we found that the overall knowledge and effectiveness scores across all participants and all 3 videos greatly exceeded our a priori criteria. It is possible that HIVRR knowledge was high at baseline in this population. Unfortunately, we did not conduct pretests of our participants' knowledge in this pilot study.

 To get a sense of behavior change possibly resulting from the mHIVRR intervention, we compared urine results for 1 month before and 1 month after the intervention period. We found no change in percent heroin-positive and percent cocaine-positive urines. The lack of impact may have been due to the short duration of the intervention and the fact that baseline drug use in our participants was already low at the start of the trial. 

 During the same pre- and post-intervention time periods, we compared EMA event-contingent entries initiated during drug craving or use events to determine if route of administration of heroin and/or cocaine changed pre- and post-intervention. We found no change in real-time self-reported route of administration. Again, this may be related to the short intervention length and low rates at baseline of HIV risk behaviors.

Limitations of the study include the lack of pre-intervention knowledge assessment and lack of random assignment to an intervention and a control group. Although we were unable to comment on relative knowledge gain as a result of the intervention due to the lack of pre-intervention knowledge assessment, we did find high rates of absolute HIVRR knowledge post-intervention. Our future studies will include randomization into control and intervention groups, additional video modules, longer intervention lengths, and pre-intervention knowledge assessment.

This project was unique because in addition to delivering the HIVRR video education via smartphone, we also conducted all acceptability and knowledge assessments on the smartphone in the participant's natural environment. Previous studies describing HIVRR video-based smartphone interventions conducted assessments by ACASI [21, 22] or by smartphone in a laboratory setting with a researcher present [33]. Another advantage of this study was that the video modules were loaded onto the smartphones and did not rely on streaming, which minimized the possibility of technical issues with syncing, reception, and network coverage [21]. Additionally, this obviated the need for an expensive data plan. To our knowledge, only one previous study utilized smartphone-delivered video-based HIVRR education delivery, but this study used computer-based ACASI acceptability assessments and streamed videos [21]. A third advantage of this study was that the video module initiation was done by the participant at a time he/she deemed convenient and private and was not prompted. Other studies have used email to prompt video initiation and have had difficulties with unreceived or accidentally deleted messages [21]. Therefore, although 24/7 viewing of the videos was possible (and encouraged) in those studies, viewing was only possible if the participant actually received the email and had no issues with video streaming, which was not always the case. In our study, participant-initiated viewing avoided the reliance on email and also allowed participants to view the video module several times during the week of release and thereafter, which maximized HIVRR knowledge acquisition. A final strength of our study was the 100% equipment recovery rate, which was likely related to providing compensation for device return and the fact that our participants were participating in a larger EMA and GMA study that required continued smartphone use. While the equipment recovery rate of 100% occurred in the small mHIVRR study sample, we also have a smartphone recovery rate of 90% in the larger parent study of over 130 individuals to date, which indicates that even in larger samples, there was minimal equipment loss.

## 5. Conclusions

Video-based mHIVRR education delivered via smartphone is acceptable, feasible and may increase HIV/STD risk-reduction knowledge. Future studies, with pre-intervention assessments of knowledge and random assignment, are needed to confirm these findings. 

## Figures and Tables

**Table 1:** Acceptability questions across all 3 videos (*n* (%)).

Functional acceptability		Methadone (MTD), n = 11	Buprenorphine (BUP), n = 13	Total	*P* value
(1) How easy was it to *play* this module? Median (IQR) 1 (1,1)	1—very easy	53 (64%)	64 (89%)	117 (75%)	*P* < 0.005
	2	16 (19%)	7 (10%)	23 (15%)	
	3	7 (8%)	1 (1%)	8 (5%)	
	4	4 (5%)	0 (0%)	4 (3%)	
	5—very hard	3 (4%)	0 (0%)	3 (2%)	

(2) How easy was it to *see and hear* this module? Median (IQR) 1 (1,2)	1—very easy	52 (63%)	52 (72%)	104 (67%)	*P* = 0.172
	2	19 (23%)	14 (20%)	33 (21%)	
	3	7 (8%)	4 (6%)	11 (7%)	
	4	3 (4%)	1 (1%)	4 (3%)	
	5—very hard	2 (2%)	1 (1%)	3 (2%)	

(3) How easy was it to *understand* this module? Median (IQR) 1 (1,1)	1—very easy	56 (68%)	61 (85%)	117 (75%)	*P* = 0.007
	2	18 (22%)	11 (15%)	29 (19%)	
	3	6 (7%)	0 (0%)	6 (4%)	
	4	1 (1%)	0 (0%)	1 (1%)	
	5—very hard	2 (2%)	0 (0%)	2 (1%)	

Educational acceptability		Methadone (MTD)	Buprenorphine (BUP)	Total	*P*-value

(4) How effective was this module in teaching you *something new* about HIV/AIDS? Median (IQR) 1 (1,2)	1—very effective	48 (58%)	60 (83%)	108 (70%)	*P* < 0.005
	2	16 (19%)	6 (8%)	22 (14%)	
	3	13 (16%)	2 (3%)	15 (10%)	
	4	4 (5%)	2 (3%)	6 (4%)	
	5—not at all effective	2 (2%)	2 (3%)	4 (2%)	

(5) How effective was this module in *reminding you* of things you knew about HIV/AIDS but had not been thinking about? Median (IQR) 1 (1,2)	1—very effective	54 (65%)	60 (83%)	114 (74%)	*P* = 0.021
	2	24 (29%)	7 (10%)	31 (20%)	
	3	4 (5%)	2 (3%)	6 (4%)	
	4	1 (1%)	1 (1%)	2 (1%)	
	5—not at all effective	0 (0%)	2 (3%)	2 (1%)	

(6) How much did this module *apply to situations in your life*? Median (IQR) 2 (1,4)	1—very applicable	19 (23%)	31 (43%)	50 (32%)	*P* = 0.722
	2	30 (36%)	5 (7%)	35 (23%)	
	3	15 (18%)	7 (10%)	22 (14%)	
	4	11 (13%)	7 (10%)	18 (12%)	
	5—not at all applicable	8 (10%)	22 (30%)	30 (19%)	

(7) How *entertaining* was this module? Median (IQR) 2 (1,3)	1—very entertaining	26 (31%)	50 (70%)	76 (49%)	*P* < 0.005
	2	17 (20%)	11 (15%)	28 (18%)	
	3	28 (34%)	6 (8%)	34 (22%)	
	4	12 (15%)	2 (3%)	14 (9%)	
	5—very boring	0 (0%)	3 (4%)	3 (1%)	

(8) How appropriate was the *length* of the module?	1—nowhere near long enough	2 (2%)	5 (7%)	7 (4%)	*P* = 0.408
	2—not quite long enough	15 (18%)	0 (0%)	15 (10%)	
	3—just about right	58 (70%)	65 (90%)	123 (80%)	
	4—a little too long	6 (7%)	2 (3%)	8 (5%)	
	5—much too long	2 (2%)	0 (0%)	2 (1%)	

Comparative acceptability		Methadone (MTD)	Buprenorphine (BUP)	Total	*P*-value

(9) Would it be better if the information was in a *booklet instead* of on the smartphone?	No	76 (92%)	62 (86%)	138 (89%)	*P* = 0.312
	Yes	7 (8%)	10 (14%)	17 (11%)	

(10) Would it be better if the information was on a full-size *computer instead* of on the smartphone?	No	60 (72%)	50 (69%)	110 (71%)	*P* = 0.725
	Yes	23 (28%)	22 (31%)	45 (29%)	

(11) Would it be better if the information was *text instead* of video?	No	71 (86%)	70 (97%)	141 (91%)	*P* = 0.012
	Yes	12 (14%)	2 (3%)	14 (9%)	

Questions 1–8 were analyzed using the Wilcoxon rank sum test for ordinal variables, and Questions 9–11 were analyzed using Fisher's exact test for dichotomous variables and small sample sizes.

**Table 2:** Knowledge/effectiveness questions and scores.

Module question no.	Question	Correct responses *n* (%)
1-1	Do you always have symptoms with a sexually transmitted disease (STD)?	45 (88%)
1-2	Do condoms come in different sizes, shapes, styles, colors, and flavors?	49 (96%)
1-3	Do you need to squeeze the tip of the condom when placing it on?	43 (84%)
	Total (%) correct for module 1	**137 (88%)**
2-1	Do you need to use a *new* condom every time you have sex from start to finish?	55 (100%)
2-2	Are body lotions, oils, or Vaseline good products to use with latex condoms?	51 (93%)
2-3	Should you remove a condom when the penis is still erect?	46 (84%)
	Total (%) correct for module 2	**152 (92%)**
3-1	Can having too much to drink or being high increase your risk for STDs and HIV?	43 (88%)
3-2	Can you tell if someone has an STD or HIV just by looking at them?	47 (96%)
3-3	Do condoms protect you against STDS, HIV, and pregnancy?	48 (98%)
	Total (%) correct for module 3	**138 (94%)**

	Total (%) correct for all 3 modules	92%

**Table 3:** Demographic and drug use characteristics at baseline (*n* = 24).

	Methadone (MTD) *n* = 11	Buprenorphine (BUP) *n* = 13	*P*-value
Male (*n* (%))	9 (81%)	11 (85%)	n.s.
African American (*n* (%))	8 (73%)	7 (54%)	n.s.
Age (mean ± SD)	43.5 ± 8.7	40.5 ± 6.8	n.s.
Education in years (median (IQR))	12 (12,12)	12 (11, 12)	n.s.
Married (*n* (%))	0 (0%)	3 (30%)	n.s.
Days paid for work in last 30 (mean ± SD)	3.6 ± 8.4	9.2 ± 8.9	n.s.
Usual full-time employment (*n* (%))	4 (44%)	3 (30%)	n.s.
Days cocaine use in last 30 (median (IQR))	0 (0, 15)	0.5 (0, 4)	n.s.
Days heroin use in last 30 (mean ± SD)	19.6 ± 10.4	13.5 ± 10.4	n.s.
Days other opiate use in last 30 (mean ± SD)	4.1 ± 6.0	17.7 ± 11.6	<0.005
Days alcohol use in last 30 (median (IQR))	0 (0, 0)	1 (0, 3)	n.s.
Days alcohol intox last 30 (median (IQR))	0 (0, 0)	0.5 (0, 3)	n.s.
Years cocaine use (median (IQR))	10 (1,20)	1 (0, 7)	n.s.
Years heroin use (mean ± SD)	17.9 ± 12.0	12.3 ± 9.3	n.s.
Years other opiate use (median (IQR))	0 (0,2)	1 (0,7)	n.s.
Years alcohol use (median (IQR))	4 (0,4)	1 (0,13)	n.s.

Categorical variables (gender, ethnicity, marital status, and employment status) were analyzed using Fisher's exact test. Continuous variables were analyzed using student *t*-tests (normally distributed) and Wilcoxon rank sum tests (nonnormally distributed).
